# Zone-wise occupancy schedules developed using Time Use Survey data for building energy performance simulations

**DOI:** 10.1016/j.dib.2023.109453

**Published:** 2023-07-26

**Authors:** Divyanshu Sood, Ibrahim Alhindawi, Usman Ali, Donal Finn, James A. McGrath, Miriam A. Byrne, James O'Donnell

**Affiliations:** a School of Mechanical and Materials Engineering and UCD Energy Institute, University College Dublin, Ireland; b School of Physics & Ryan Institute's Centre for Climate and Air Pollution Studies, University of Galway, Ireland; c Department of Experimental Physics, Maynooth University, Maynooth, County Kildare, Ireland

**Keywords:** Occupancy schedules, Occupant behaviour, Building energy performance simulation, Time use survey data

## Abstract

The occupancy profile dataset presented in this study leverages publicly available UK Time Use Survey (TUS) 2014–15 data to evaluate the impact of occupancy on energy consumption at various spatial and temporal scales using multi-scale archetypes. Constructing this occupancy dataset includes conversion, categorisation, extraction and analysis processes. The resulting dataset (in .csv) format represents realistic day-wise zone-level occupancy availability schedules that account for the effect of the type of dwelling, the number of occupants, the month of the year and the day of the week. A total of 5,376 occupancy profiles were extracted, representing a large number of dwellings. These profiles demonstrate the realistic behaviour of occupants’ availability in dwellings. These profiles allow us to gain valuable insights into the energy usage patterns in dwellings based on the realistic behaviour of occupants, leading to more accurate and context-specific energy assessments.

**Specification Table**

**Table:** 

Subject	Engineering
Specific subject area	Building Energy Performance Simulation (BEPS) and occupancy behaviour
Type of data	Occupancy availability schedules in .csv format
How data were acquired	The primary data for this study were collected in the UK through surveys known as the United Kingdom Time Use Survey (TUS) data [1], which is publicly available. The TUS data captures qualitative information about various activities performed by residents in their dwellings, including sleeping, watching TV, cooking, and eating, among others. However, the TUS data, in its current form, is not suitable for building performance simula-tions. To overcome this limitation, the activities recorded in the TUS data were transformed into occupants’ availability schedules using Python pro-gramming. Several scripts extract the required data in a more useful format, specifically values ranging from 0 to 1. These processed data are now directly usable with EnergyPlus for conducting building performance simulations.
Data format	Cleaned and formatted data in Comma-separated values (.csv)
Description of data collection	From the publicly available TUS data [1], we processed the data to extract day-wise occupancy profiles that are representative of a large number of dwellings. The dataset includes an activity diary that records occupants’ activities at 10-minute intervals. To develop the occupancy schedules, we utilized information such as the activities performed at 10-minute intervals, with whom the activity is being performed, dwelling types, number of occupants, months, and days. The developed profiles are based on various attributes which impact the occupants’ behaviour. This approach helps to account for variations in occupants’ schedules that can be attributed to these factors.
Data source location	All primary data were collected from the UK TUS data [1]
Data accessibility	Repository name: Zone-wise occupancy profiles (Mendeley Data) Direct URL to data: https://data.mendeley.com/datasets/vr2fmxpczd/1
Related research article	[2] Sood, Divyanshu, Ibrahim Alhindawi, Usman Ali, James A. McGrath, Miriam A. Byrne, Donal Finn, and James O'Donnell. “Simulation-based eval-uation of occupancy on energy consumption of multi-scale residential building archetypes.” Journal of Building Engineering (2023): 106,872. https://doi.org/10.1016/j.jobe.2023.106872

## Value of the Data

• The data represents realistic day-wise schedules at room level for residential dwellings. These profiles consider the influence of dwelling type, the number of occupants, the month of the year, and the day of the week on the availability of occupants. Considering these attributes enables a deeper understanding of how different factors influence occupancy patterns and behaviour.
• The primary data used for this study is in qualitative form, which is not directly suitable for building energy simulations. The methodology developed in this study effectively transforms the primary qualitative data into a quantifiable format, extracting pivotal information and generating profiles conducive to building energy performance simulations in EnergyPlus.
• The developed profiles represent occupancy patterns of a large number of dwellings. Consequently, these realistic profiles serve as a valuable resource for creating robust archetypes, which are instrumental in urban building energy modelling. By incorporating these profiles into building energy models, we can effectively reduce the discrepancies between predicted energy consumption and actual measured energy usage resulting from occupancy behaviour. This improvement addresses the challenges associated with accurately capturing the impact of occupancy behaviour on energy consumption, enhancing the reliability and accuracy of energy simulations.
• These occupancy profiles serve as a valuable tool for analysing and understanding the dynamics of occupancy behaviour in relation to various contextual factors. They enable researchers to investigate the influence of dwelling type, number of occupants, and temporal factors on building energy performance. By incorporating these profiles into building energy models, we gain valuable insights into the energy usage patterns of different types of dwellings, leading to more accurate and context-specific energy assessments.
• These occupancy profiles can provide valuable insights for policymakers, local authorities, and urban planners to make informed decisions based on actual data. This approach ensures that energy-related decisions and interventions are tailored to the specific needs and behaviour of occupants, leading to more targeted and impactful outcomes.

## 1 Objective

This research article utilizes the publicly available UK TUS dataset [1] to develop day-wise occupancy schedules to evaluate the impact of occupancy on multi-scale residential building archetypes to estimate energy consumption at various spatial and temporal scales. By publishing the occupancy dataset alongside the article, the research becomes more transparent, allowing for verification and reproducibility of the findings. The primary objective of the research article is to present a user-friendly framework for extracting occupancy schedules from TUS data. This framework aims to provide an easily implementable method for obtaining occupancy schedules based on the dataset. This data article's primary purpose is to document the developed occupancy schedules. This documentation ensures that the schedules can be readily employed for building energy performance simulations. This rich dataset offers valuable insights into the temporal dynamics of household activities, enabling researchers to gain a deeper understanding of human behaviour in the context of energy consumption and resource management.

## 2 Data Description

**Fig. 1:**
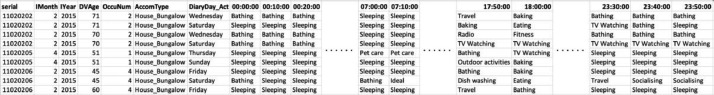
Snippet from the TUS data to provide an initial impression of the data [1].

The initial examination of the UK Time Use Survey (TUS) 2014–15 dataset provides valuable insights into the diverse range of activities performed by household occupants. This dataset encompasses 16,550 diary days completed by respondents who participated in two 24-hour diary sessions. Each diary entry captures occupants’ activities at 10-minute intervals (refer to Fig. 1). The dataset comprises data from a total of 11,860 sampled households, resulting in 4238 household interviews. Among the interviewed households, there were 10,208 eligible respondents, of whom 9388 provided answers during individual interviews and/or completed the 16,550 diary days. In addition to activity records, the diary includes supplementary information such as housing type, the number of adults and children residing in the household, and the month and day of the week in which the diary was completed. The days of the week were coded numerically, with 1 representing Sunday and a consecutive pattern for the remaining days. Furthermore, each house was assigned a unique serial number, ensuring anonymity while facilitating the identification of the total number of respondents within a given household. The detailed documentation and structure of the TUS 2014–15 dataset provide a solid foundation for conducting comprehensive analyses of occupancy patterns and their implications for various domains, such as building energy modelling.

**Fig. 2:**
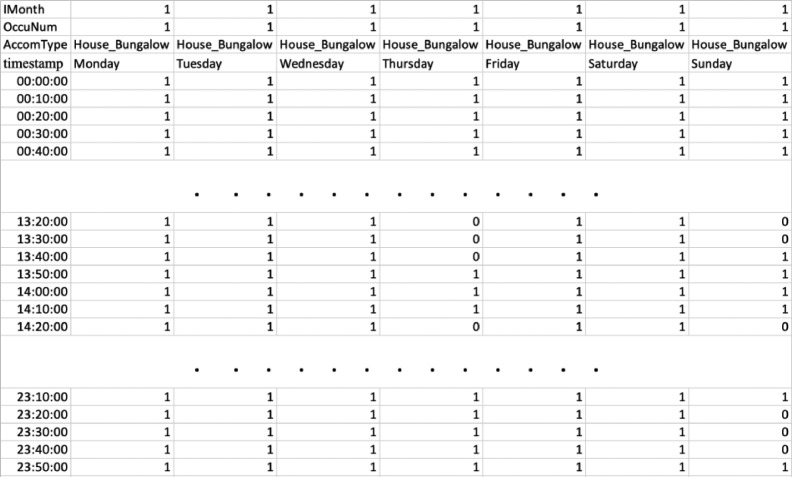
Snippet from the developed occupancy schedules data to provide an impression of the profiles.

This paper utilizes the TUS data and processes it to create daily occupancy schedules. In this study, the primary focus is not on providing the TUS data itself but rather on creating realistic room-level occupancy schedules that can be directly utilized for building energy performance simulations. The aim is to develop accurate representations of how occupants utilize rooms throughout the day, considering activities such as sleeping, cooking, eating, bathing, and more. By utilizing the TUS data as a basis, we have processed and analysed it to extract meaningful patterns and create these occupancy schedules (Fig. 2) in .csv format, which can be directly used for building energy performance simulations. IMonth represents the month of the year starting with January (1), February (2) and so on. OccuNum is the number of occupants in a dwelling. AccomType indicates the type of dwellings. The profiles are developed at 10-minute intervals. We made an assumption that certain types of apartment-style dwellings, including top, mid, and ground floor apartments, maisonettes, and basement dwellings, share similar characteristics with flats or maisonettes. As a result, the occupancy profiles developed for flats or maisonettes are also applicable to these various apartment types. Similarly, for house-type dwellings such as semi-detached, detached, and terraced houses, we utilized the profiles developed for houses or bungalows. These occupancy profiles play a crucial role in comprehending the impact of different parameters, such as dwelling type, number of occupants, and specific days and months, on occupancy behaviour. To capture the variations in occupancy behaviour throughout the week, we developed weekly profiles that represent an entire month. By employing data statistics.mode() analysis, we identified the most frequently occurring daily activities within the dataset. Consequently, these weekly average profiles are utilized to rep- resent occupancy behaviour for the corresponding month. These schedules offer valuable insights into the occupancy behaviour within buildings, allowing for more precise simulations of energy consumption and overall building performance. By sharing these schedules with the public, the study aims to facilitate the integration of realistic occupancy patterns into building simulations, enabling more accurate assessments of energy usage and the effectiveness of energy-saving strategies.

There are four folders Apartment 1occupant, Apartment 2occupants, Apartment 3occupants, and Apartment 4occupants. Each of these folders represents apartments based on the number of occupants. Within each sub-folder, there are four additional sub-folders named Bathroom, Bedroom, Kitchen, and Living Room. These sub-folders correspond to the different areas or rooms within each apartment. In each of these room-specific sub-folders, there are a total of 84 CSV files in each sub-folder. Each file is named in the format Number of occupants month of the year day of the week. This naming convention allows for easy identification and organization of the data contained within these files.

Similarly, there are four more folders for the house-type dwelling, named House 1occupant, House 2occupants, House 3occupants, and House 4occupants. These sub-folders represent differ- *ent* types of houses based on the number of occupants they can accommodate. Each of these sub- folders contains three additional sub-folders named Bungalow, Detached House, and Semi-Detached House. These sub-folders represent different types of houses based on their architectural style.

Within each of these sub-folders, there are four sub-sub-folders named Bathroom toilet, Bedroom (1–4), Kitchen dining, and Living Room These sub-sub-folders correspond to specific areas or rooms within each house. In each sub-sub-folder, there are a total of 84 CSV files. The naming convention for each file follows the format Number of occupants month of the year day of the week. This systematic naming scheme facilitates easy identification and organization of the data contained in these files.

## 3 Experimental Design, Materials and Methods

This paper is motivated by the lack of zone-wise occupancy schedules and aims to make the dataset publicly available to the research community. The primary data used to develop occupancy profiles in this study was obtained from the UK TUS data [1]. By leveraging the TUS data, the study created day-wise room-level occupancy schedules that represent a wide range of dwellings. The TUS data provide qualitative information about activities, companions, dwelling types, and the number of occupants. However, this information cannot be directly used for building energy simulations, which require input data in a binary format (0 or 1). The originality lies in the easy-to-implement methodology for the conversion of qualitative time-use data into a quantitative form compatible with EnergyPlus, allowing direct usage for building energy performance simulations. This approach involves categorizing activities, assigning them to specific zones within the dwelling, and determining the presence or absence of occupants at 10-minute intervals. Various factors, including dwelling type, number of occupants, the month of the year, and day of the week, were considered to capture their influence on occupancy behaviour. This method's structured and systematic framework allowed for the integration of occupancy data into energy simulations, leading to more accurate predictions of energy consumption in buildings. This chapter provides a detailed explanation of the entire process, from data collection to profile extraction, including the algorithm employed for each stage.

### 3.1 Preparation and cleaning of the TUS data

The development of occupancy schedules in this study relies on the UK Time Use Survey (TUS) data, which is publicly available [1]. The TUS data was recorded using a numeric format, where each number corresponds to a specific activity performed by the occupants. The numeric entries were converted into the activity name using the information in the TUS dictionary. However, it is important to note that the TUS data may contain entries with missing or incomplete information regarding occupancy activities. To ensure data consistency throughout the analysis, rows with incomplete or non-applicable entries were removed from the dataset. In order to capture the presence or absence of residents in a dwelling based on their activities, the recorded activities were categorized into two groups: 1) Outdoor Activities (OA), and 2) At home. This categorization was based on the information provided by the TUS dictionary. For further details and the specific classification of activities, please refer to Table 1. By categorizing the activities in this manner, we can effectively distinguish between the times when occupants are engaged in activities outside the home and when they are present at home.

**Table 1:** Categorisation of activities into “Outdoor Activities” and “At home” based on the nature of activity.

Outdoor Activities (OA)	At home
Travel related to unspecified time use	Sleep
Travel related to personal business	Sleep: In bed not asleep
Travel to/from work	Sleep: Sick in bed
Travel in the course of work	Eating
Travel to home	Unspecified personal care
Travel to other place	Wash and dress
Travel related to education	Other personal care
Travel to/ from education	Study: Homework
Travel related to shopping	Free time study
Travel related to services	Unspecified household and family care
Travel other than education	Unspecified food management
Travel escorting an adult	Food preparation and baking
Travel related to work	Dish washing
Travel related to meetings	Preserving
Travel related to households	Other specified food management
Travel related to religious activities	Unspecified household upkeep
Travel related to participatory activities	Cleaning dwelling
Travel to visit friends/relatives	Cleaning yard
Travel related to social activities	Heating and water
Travel for entertainment and culture	Arranging household goods and materials
Travel related to other leisure	Disposal of waste
Travel related to physical exercise	Other or unspecified household upkeep
Travel related to hunting & fishing	Unspecified making and care for textiles
Travel related to exercise & fishing	Laundry
Travel related to gambling	Ironing
Travel related to changing locality	Making and care for textiles
Travel to holiday base	Gardening
Travel for day trip/just walk	Tending domestic animals
Other specified travel	Caring for pets
Unspecified employment	Walking the dog
unspecified main job	Other specified gardening and pet care
Working time in main job	Unspecified construction and repairs
Coffee and other breaks in main job	House construction and renovation
Working time in second job	Repairs of dwelling
Coffee and other breaks in second job	Repairing and maintaining equipment
activities related to employment	Woodcraft sculpture and pottery
Lunch break	Other specified repairing equipment
Other activities related to employment	Vehicle maintenance
Activities related to job seeking	Other specified construction and repairs
Other activities related to employment	Personal services
study school or university	Other specified shopping and services
activities related to school or university	Household management not using the internet
Classes and lectures	clothing via the internet
Unspecified shopping and services	household management using the internet
Unspecified shopping	Shopping via the internet
Shopping mainly for food	Food via the internet
Shopping mainly for clothing	goods and services via the internet
Shopping mainly related to accommodation	Shopping mass media via the internet
Shopping car boot sales or antique fairs	Shopping entertainment via the internet
Window shopping or other shopping as leisure	Banking and bill paying via the internet
Other specified shopping	household management using the internet
Commercial and administrative services	Unspecified childcare
Accompanying household member to hospital	Unspecified physical care & supervision of a child
Unspecified volunteer work and meetings	Feeding the child
Unspecified organisational work	unspecified physical care & supervision of a child
Work for an organisation	Teaching the child
Volunteer work through an organisation	Reading playing and talking with child
Other specified organisational work	Accompanying child
Shopping and services as help to households	Other or unspecified childcare
Help households in employment and farming	Unspecified help to a non-dependant
Meetings	Physical care of a non-dependant
Religious activities	Other specified help to a non-dependant
Other specified participatory activities	Unspecified help to a dependant
Unspecified social life and entertainment	Physical care of a dependant
Unspecified social life	Accompanying a dependant adult ic
Socialising with family	Other specified help to adult
Visiting and receiving visitors	informal help to households
Celebrations	Food management
Other specified social life	Household upkeep
Unspecified entertainment and culture	Gardening and pet care
Cinema	Construction and repairs
Unspecified theatre or concerts	Unspecified childcare
Plays musicals or pantomimes	supervision of child
Opera operetta or light opera	Teaching non-coresident child
Concerts or classical music	Reading to non-coresident child
Live music	Accompanying non-coresident child
Dance performances	Physical care of own child
Other specified theatre or concerts	Reading playing & talking to own child
Art exhibitions and museums	Accompanying own non-coresident child
Unspecified library	Other specified childcare
Borrowing books from a library	Unspecified help to an adult
Reference to books within a library	supervision of an adult
internet in library	Accompanying an adult to another household
Using computers in library	Other help to an adult
Reading newspapers in library	Other informal help to another household
Other specified library activities	Other specified informal help
Sports events	Unspecified participatory activities
Other entertainment and culture	Telephone conversation
Visiting a historical site	Unspecified hobbies and computing
Visiting wildlife site	Unspecified arts
Visiting a botanical site	Unspecified visual arts
Visiting a leisure park	Painting or graphic arts
Visiting an urban playground	Making videos taking photographs
Other entertainment or culture	Other specified visual arts
Resting - Time out	Unspecified performing arts
Unspecified sports	Singing or musical activities
Unspecified physical exercise	Other specified performing arts
Walking and hiking	Literary arts
Taking a walk or hike	Other specified arts
Other walk or hike	Unspecified hobbies
Jogging and running	Collecting
Biking skiing and skating	Correspondence
Biking	Other arts and hobbies
Skiing or skating	Computing - programming
Unspecified ball games	Unspecified information by computing
Indoor pairs or doubles games	Information searching on the internet
Indoor team games	Other specified information by computing
Outdoor pairs or doubles games	Unspecified communication by computer
Outdoor team games	Communication on the internet
Other specified ball games	Other specified communication by computing
Gymnastics	Unspecified other computing
Fitness	Skype or other video call
Unspecified water sports	Other specified computing
Swimming	Unspecified games
Other specified water sports	Solo games and play
Other specified physical exercise	Unspecified games and play with others
Hunting and fishing	Chess and bridge
Other specified productive exercise	Computer games
Unspecified sports related activities	Gambling
Activities related to sports	Other specified games
Activities related to productive exercise	Unspecified mass media
	Unspecified reading
	Reading periodicals
	Reading books
	Other specified reading
	Unspecified TV video or DVD watching
	Watching a film on TV
	Watching sport on TV
	Other specified TV watching
	Unspecified video watching
	Watching a film on video
	Watching sport on video
	Other specified video watching
	Unspecified listening to radio and music
	Unspecified radio listening
	Listening to music on the radio
	Listening to sport on the radio
	Other specified radio listening
	Listening to recordings

As outdoor activities have no direct influence on building energy performance, the primary focus of this study is on activities conducted within dwellings. To streamline the analysis, the activities categorized as “at home” were further grouped into fewer categories based on their descriptions as provided in the TUS dictionary. For instance, activities such as sleep, sleep: in bed but not asleep, and sleep: sick in bed were all consolidated under the category of Sleeping. This grouping process aimed to provide a more concise representation of occupancy profiles by reducing the total number of activities in the dataset to 19. By consolidating activities into broader categories, we achieve a more manageable and meaningful classification that captures the essential aspects of occupancy behaviour. For a comprehensive overview of the activity grouping, please refer to Table 2.

**Table 2:** Grouping “At home” activities into sub-groups.

“At home” activities	Final activity groups
Sleep	
Sleep: In bed not asleep	Sleeping
Sleep: Sick in bed	

Toilette	
Bathing	Using bathroom
Brushing	

Cooking/Baking	Cooking

Eating	Eating

Laundry	Laundry

Unspecified other personal care	
Wash and dress	Personal care
Personal services	
Other specified personal care	

Study: Homework	
Study: related to school or university	Study
Free time study	

Unspecified food management	
Food preparation and baking	Food Management
Preserving	
Other specified food management	
Food management as help to other households	
Dish washing	Dish washing

Unspecified household upkeep	
Cleaning dwelling	
Cleaning yard	
Heating and water	Household Management
Arranging household goods and materials	
Disposal of waste	
Other or unspecified household upkeep	
Ironing	
Gardening	
Unspecified making and care for textiles	
Household management not using the internet	

Unspecified TV video or DVD watching	
Watching a film on TV	
Watching sport on TV	
Other specified TV watching	
Unspecified video watching	
Watching a film on video	
Watching sport on video	
Other specified video watching	TV/Music/Radio
Unspecified listening to radio and music	
Unspecified radio listening	
Listening to music on the radio	
Listening to sport on the radio	
Other specified radio listening	
Listening to recordings	
Singing or other musical activities	

Computing - programming	
Unspecified information by computing	
Information searching on the internet	
Other specified information by computing	
Unspecified communication by computer	
Communication on the internet	
Other specified communication by computing	
Unspecified other computing	
Skype or other video call	
Other specified computing	
Computer games	
Unspecified mass media	
Unspecified hobbies games and computing	
Other specified shopping and services	Using computer
Shopping for and ordering clothing via the internet	
Unspecified household management using the internet	
Shopping unspecified goods and services via the internet	
Shopping for and ordering food via the internet	
Shopping goods and services via the internet	
Shopping for and ordering mass media via the internet	
Shopping for and ordering entertainment via the internet Banking and bill paying via the internet	
Other specified household management using the internet	

Unspecified games	
Solo games and play	
Unspecified games and play with others	
Billiards pool snooker or petanque	
Chess and bridge	
Other specified parlour games and play	
Gambling	Entertainment
Other specified games	
Unspecified participatory activities	

Unspecified arts	
Unspecified visual arts	
Painting drawing or other graphic arts	
Other specified visual arts	
Unspecified performing arts	
Other specified performing arts Literary arts	
Other specified arts	
Unspecified hobbies	Hobbies
Collecting	
Correspondence	
Other specified or unspecified arts and hobbies	

Telephone conversation	On Phone

Unspecified childcare as help to other households	
Physical care and supervision of child	
Teaching non-coresident child	
Reading playing & talking to non-coresident child	
Accompanying non-coresident child	
Physical care and supervision of own child	Childcare
Reading playing & talking to own non-coresident child	
Accompanying own non-coresident child	
Other specified childcare as help to other household	
Reading playing and talking with child	
Accompanying child	
Other or unspecified childcare	
Unspecified childcare	
Unspecified physical care & supervision of a child	
Feeding the child	
Other and unspecified physical care & supervision of a child	
Teaching the child	

Physical care and supervision of an adult	
Accompanying an adult as help to another household	
Other specified help to an adult member	
Other specified informal help to another household	
Other specified informal help	
Unspecified help to a non-dependant	
Physical care of a non-dependant	
Other specified help to a non-dependant adult	
Unspecified help to a dependant adult household member	
Physical care of a dependant adult	
Accompanying a dependant adult	
Other specified help to a dependant adult	Helping Adult
Unspecified informal help to other households	
Household upkeep as help to other households	
Gardening and pet care as help to other households	

Unspecified reading	
Reading periodicals	
Reading books	Reading
Other specified reading	

Tending domestic animals	
Caring for pets	Pet care
Walking the dog	
Other specified gardening and pet care	

Following the grouping of activities within the “At home” category into a refined set of activity types (referred to as Final activity groups), these activities are further assigned to different zones based on their nature and typical location. The TUS dataset provides valuable information regard ing the individuals involved in each activity, aiding in the determination of appropriate zones for each activity. To maintain consistency throughout the analysis, several assumptions were made prior to converting the TUS data into occupancy profiles, as detailed in work by [2]. By assigning activities to specific zones based on their characteristics and typical locations, we enhance the accuracy and realism of the occupancy profiles. These profiles capture the spatial distribution of activities within dwellings, enabling more realistic simulations of energy consumption and performance. The assumptions made during the conversion process ensure a standardized approach and facilitate reliable comparisons across different building typologies and occupancy scenarios. The incorporation of these occupancy profiles into energy simulations provides valuable insights for optimizing building design, energy management strategies, and resource allocation in the built environment.

1. The activities in the dataset were categorized based on their nature and the corresponding room or location where they typically take place. This categorization of activities and their respective locations is presented in Table 3.

**Table 3:** Determining the location of activity based on the type of activity and typical location of the activity can be performed [2].

Bedroom	Bathroom	Kitchen Dining	Living Room
Sleeping Household Management Personal care Study TV/Music/Radio On Phone Childcare Helping Adult Reading	Household Management Using bathroom	Cooking Eating Laundry Food Management Dish washing	Study Household Management TV/Music/Radio Using Computer Entertainment Hobbies On Phone Childcare Helping adult Reading Pet care
2. Activities that take place outside of the dwelling, including activities such as going to the gym, running, travelling, engaging in social activities, participating in sports, administrative work, banking, office work, watching movies or concerts, shopping, swimming, and taking office breaks, are grouped together as “Outdoor Activities” or “OA.”

**Fig. 3:**
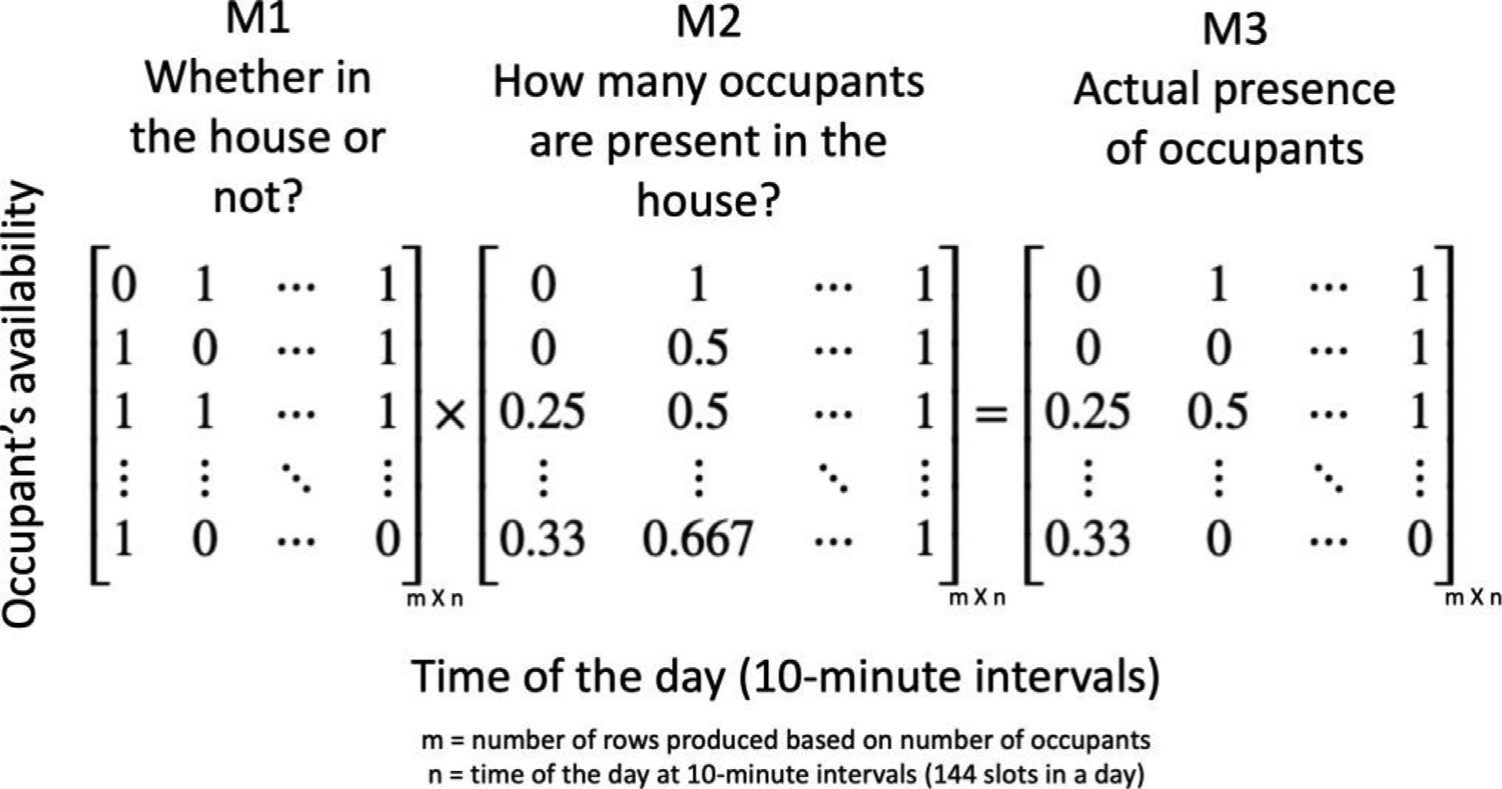
Element-wise array multiplication to identify the actual presence of occupants in households with two or more occupants [2].

To extract occupancy profiles, the process involved the creation of two arrays through element- wise array multiplication, as depicted in Fig. 3. This method, as described in work by [2], determined the presence of occupants in different zones within households consisting of two or more occupants. The first array, denoted as M1, represents the location of each occupant and indicates their presence (1) or absence (0) in specific zones such as the bedroom, bathroom, kitchen dining, or living room. The determination of occupant location was based on their activities and the corresponding zones where those activities typically occurred (refer to Table 3). For instance, in a dwelling occupied by a single individual, if the occupant was present in the common room, a value of 1 was assigned to the common room zone, while the other zones were assigned a value of 0. Similarly, for households with 2, 3, and 4 occupants, the zones where occupants were present were assigned a value of 1, while the remaining zones were assigned a value of 0.

The second array, denoted as M2, was created to represent the total number of occupants present in a dwelling at a given moment, irrespective of their specific location within the house. The TUS dataset provided information on the number of occupants involved in activities with various companions, such as being alone, with a mother, with a spouse, with a father, or with a child, at 10-minute intervals. Seven distinct datasets were available, each corresponding to a specific type of companionship. These individual datasets were combined to determine the total number of occupants present in the dwelling at each 10-minute interval. The combined array was then divided by the total number of occupants in the dwelling to convert the data into a format compatible with EnergyPlus. For example, in a dwelling with 2 occupants, only the dataset containing 2 occupants was considered and divided by 2 after summing up all datasets involving 2 occupants. The resulting element-wise array multiplication produced the actual occupancy availability of occupants in different zones, denoted as M3. The values in this matrix ranged from 0 to 1, indicating the extent of occupancy in each zone. While the composition of M1 varied with respect to the zone, M2 remained constant as it provided information on the total number of occupants present in the dwelling for a given number of occupants.

This array-based approach allows for a systematic representation of occupancy patterns and facilitates the extraction of meaningful occupancy profiles. By capturing the spatial distribution of occupants within households, we gain valuable insights into the utilization of different zones and the corresponding implications for energy consumption and building performance. These occupancy profiles contribute to the development of realistic simulation-based archetypes and enhance the accuracy of energy simulations and performance assessments.

### 3.2 Categorisation and extraction of occupancy profiles

The categorization and extraction of data are seamlessly integrated into a single step. This step involves classifying the data based on various attributes identified within the dataset. The classification process begins by categorizing the data according to the type of dwelling, followed by the number of occupants, the month of the year, and the day of the week (as depicted in Algorithm 1). By systematically grouping the data based on these attributes, we gain valuable insights into how different factors influence occupancy behaviour. The classification process starts by grouping the data based on dwelling type and the number of occupants. Subsequently, the data is further sorted based on the month and day of the week. This multi-level classification approach enables a com- prehensive examination of how different attributes contribute to variations in occupancy patterns. Once the data classification is completed, the statistics.mode() of the data is utilized to identify the most frequently observed activities performed by occupants within each time slot. This approach ensures that the resulting occupancy profiles represent a significant number of dwellings and capture the prevailing trends in occupancy behaviour. By combining the classification and extraction processes, we achieve a robust methodology for deriving occupancy profiles that consider various attributes and their influence on occupancy patterns. These profiles contribute to a more accurate representation of real-world occupancy behaviour, facilitating improved building energy simulations and enhancing our understanding of energy performance in urban environments.

**Table:** 

**Algorithm 1** Algorithm to make data clusters based on various attributes in order to extract desired occupancy profiles from TUS data [2].

1: *i* = *HouseOrBungalow, FlatOrMaisonette*
2: **for***AccomType* = *i***do**
3: **for***OccuNum* = 1*,* 2*,* 3*,* 4 **do**
4: **for***IMonth* = 1*,* 2*,* 3 *. . .* 12 **do**
5: **for***DiaryDayAct* = 1*,* 2*,* 3 *. . .* 7 **do**
6: **end for**
7: **end for**
8: **end for**
9: **end for**
10: DiaryDayAct.mode()

## Ethics Statements

This work does not involve things like human subjects, animal experiments, and data collection from social media platforms. Moreover, there are no objections in accessing the TUS data as it is publicly available through the following link (https://ukdataservice.ac.uk/) by simply registering yourself with the project details.

## CRediT Author Statement

**Divyanshu Sood:** Conception and design of the study, Acquisition of data, Analysis and/or interpretation of data, Writing – original draft. **Ibrahim Alhindawi:** Writing – review & editing. **Usman Ali:** Acquisition of data. **James A. McGrath:** Writing – review & editing. **Miriam A. Byrne:** Writing – review & editing. **Donal Finn:** Writing – review & editing. **James O'Donnell:** Conception and design of the study, Writing – review & editing.

## Declaration of Competing Interest

The authors declare that they have no known competing financial interests or personal relationships that could have appeared to influence the work reported in this paper.

## Data Availability

Zone-wise occupancy profiles (Reference data) (Mendeley Data).
